# Will Bivalent Vaccination against COVID-19 Increase the Desire for COVID-19 Vaccination among Poles?

**DOI:** 10.3390/vaccines10101658

**Published:** 2022-10-03

**Authors:** Mateusz Babicki

**Affiliations:** Department of Family Medicine, Wroclaw Medical University, 51-141 Wroclaw, Poland; ma.babicki@gmail.com

**Keywords:** COVID-19, COVID-19 vaccination, bivalent COVID-19 vaccination

## Abstract

Tduration of persistent protection after vaccination against COVID-19 is the sum of many factors, including the used formulation, the vaccination schedule, individual predisposition, clinical status and the SARS-CoV-2 variant. (There is a tendency that?) vaccination regimens demonstrate lower levels of immunity against the currently predominant Omicron variant, which results in the need for subsequent booster doses. Thus, bivalent formulations have been recently developed to additionally target the Omicron variant. Accordingly, the purpose of this study was to assess whether bivalent vaccines would increase interest in vaccination among Poles. For this purpose, an original questionnaire distributed via the Internet and targeting Poles over the age of 18 was used. Results: The survey included 594 respondents, the vast majority of whom were women (79.3%), residents of large cities (44.7%) and those with a university education (86.3%). The average age was 36.6 ± 9.67 years. Only 48.7% of respondents had heard of bivalent vaccination against COVID-19. 408 (72.3%) respondents confirmed that if they had the choice, they would opt for the bivalent preparation. People who have already taken at least 1 booster dose are by far the most common group. Among the unvaccinated, the percentage is only 8.9%. For 190 (33.7%) of respondents, the availability of bivalent preparations will help accelerate their decision to vaccinate against COVID-19. Conclusions: Bivalent vaccination is an important part of the fight against the ongoing COVID-19 pandemic. However, it is forecasted that its implementation will not contribute significantly to the increase in vaccination-related interest rate among those previously unvaccinated or only after the basic regimen. Therefore further observations in this direction are necessary.

## 1. Introduction

More than two years have passed since the outbreak of the COVID-19 pandemic worldwide [1]. As a result of huge financial outlays and the hard work of scientists around the world, an effective vaccination against SARS-CoV-2 has been rapidly? developed. However, despite the significant effectiveness of vaccination in reducing the disease severity and mortality, the level of public acceptance in Poland remains one of the lowest in the European Union [2,3]. According to official data, less than 59% of Poles have completed the primary vaccination schedule [4]. An additional dose has been taken by 34% of eligible individuals [4]. Based on previous studies, it is known that the reason behind this is a lack of confidence in the effectiveness of the preparations used, fear of side effects, and belief in an adequate level of self-protection after COVID-19 [5]. Unfortunately, the immunity produced after COVID-19 can be inadequate and quickly disappears [6]. Likewise, the immunity produced after both the primary vaccination and a single booster dose declines over time [7,8,9].

The duration of protection is the sum of many factors including the formulation used, the vaccination schedule, individual predisposition, the patient’s clinical condition and the virus variant [10]. For example, people with compromised immune systems showed a significantly lower immune response after the vaccination [11]. Therefore, in this group of patients, the implementation of booster vaccination was crucial to maintain an adequate level of safety. Another aspect that affects the effectiveness of vaccination is the virus variant [12]. Due to ongoing changes in the structure of SARS-CoV-2, there have been reports indicating the ability of the currently predominant Omicron variant to escape the immune response, especially among those vaccinated with 1 or 2 doses [13]. Compared to unvaccinated individuals, receiving booster doses has been shown to increase the level of protection up to 40% against a severe course, and up to 86% for the fourth dose [14].

Many concerns have begun to emerge around the world as to whether taking another dose of the same product will impact the immunity and what direction should be taken regarding COVID-19 vaccination [15]. These issues may contribute to a lower vaccination-related interest rate, which is already at a relatively low level.

In 2022, the first information began to appear regarding bivalent preparations that additionally target the Omicron variant. From the communication of pharmaceutical companies involved in their development, it is known that the use of a bivalent formulation increases the level of protection against the Omicron variants, including those being the predominant i.e., BA.4 and BA.5 [16,17]. The UK is the first country that approved the formulation for widespread use on 15 August 2022 [18]. Earlier this year, Polish government assurred that bivalent formulations will appear in Q4 2022 [19].

Therefore, the purpose of this study was to assess whether the introduction of new formulations targeting the Omicron variant will increase interest in vaccination against COVID-19 in Poland. To the best of the author’s knowledge, this is the first study to assess Poles’ attitudes toward bivalent vaccination.

## 2. Materials and Methods

### 2.1. Methodology

The present study was based on the author’s questionnaire, which was distributed electronically via the social network Facebook.com. The survey was addressed to all people over the age of 18, living in Poland with access to the Internet. Participation in the survey was entirely voluntary, anonymous, and respondents did not receive financial benefits after completing the questionnaire. Before filling the survey, respondents were informed of its objectives and methodology, after which they gave their informed consent to participate in the study.

The distribution of the survey occurred during the period from 1 August to 15 August 2022, when the vaccination rate against COVID-19 was 59%, far below the European average [4]. In addition, during the distribution period of the survey, 4 doses of vaccination against COVID-19 were allowed for both people aged 60+ years and younger individuals with immunodeficiency who have not received any vaccine dose for at least 4 months [20]. By that time, the media started to report the availability of bivalent vaccination against COVID-19 in Poland [19].

The author’s questionnaire consisted of two parts. The first included an assessment of sociodemographic data including age, gender, place of residence, education level, and relationship status. It also assessed previous COVID-19 vaccination status (unvaccinated/incomplete basic regimen/basic regimen/basic regimen with booster dose), the occurrence of adverse reactions after COVID-19 and any previous vaccination. A complete vaccination regimen was defined as receiving 2 doses of Comirnaty (Pfizer/BioNTech) or 2 doses of Vaxevria (AstraZeneca) or 2 doses of Spikevax (Moderna) or 1 dose of COVID-19 Vaccine Janssen. Willingness to take a booster dose was also assessed with possible answers: as soon as possible/within a few months (up to a year)/more than a year/cannot decide/no, but will consider in the future/no, never). The next stage included questions assessing attitudes toward bivalent formulas. At the outset, it was assessed whether the respondent had ever heard of such preparations, whether he or she was afraid of such a preparation (not afraid at all/less than a monovalent preparation/as afraid as a monovalent preparation/more afraid than a monovalent preparation), and whether he or she would like to be vaccinated with a bivalent preparation. The respondent was also asked whether, in his opinion, the formulations would increase interest in vaccination against COVID-19 in Poland.

The study was conducted in accordance with the Declaration of Helsinki and approval was obtained from the Bioethics Committee for the project.

### 2.2. Statistical Analysis

The variables analyzed were qualitative and quantitative. Basic descriptive statistics were used. To compare differences between qualitative variables, the Chi2 test with Pearson’s modification was used.

All tests assumed a statistical significance level of *p* < 0.05. The analysis was performed using Statistica 13.0, StatSoft (Hamburg, Germany).

## 3. Results

### 3.1. Description of the Study Group

There were 568 respondents, of whom 4 did not agree to fill the survey. Thus, the 564 questionnaires-99.3%-were included in the final analysis. The vast majority of respondents were women (79.3%), residents of large cities (44.7%) and those with a university education (86.3%). The mean age was 36.6 ± 9.67 years (median 34 years). A detailed description of the study group is presented in Table 1.

### 3.2. Vaccination Status

Among those surveyed, 79 (14.1%) respondents still had not received a single dose of COVID-19 vaccination. The vast majority of respondents received a booster dose—69.1%. The most common formulation chosen by the respondents was Comirnaty. Of those vaccinated, by any vaccine, 56.9% of respondents had mild adverse reactions, and none of the respondents had severe adverse reactions. In the survey group of respondents, 299 (53%) respondents want to take a booster dose as soon as possible, while 99 (17.6%) said they would never take a booster dose.

A detailed summary of vaccination history is presented in Table 2.

### 3.3. Approach toward Bivalent Vaccination

Among the analyzed group, only 48.7% of respondents indicated that they have heard of bivalent preparations against COVID-19. 408 (72.3%) respondents confirmed that if they had a choice, they would opt for a bivalent preparation. They were significantly more likely to be women (76.3%; *p* < 0.001), residents of large cities (78.6%; *p* < 0.001), and those with chronic conditions (80.3%; *p* < 0.001). As many as 96.7% of respondents who want to urgently receive the next dose of vaccination also prefer the bivalent preparation. Interestingly, 76.8% of respondents who cannot decide also prefer the bivalent preparation. Among those not yet vaccinated, only 8.9% would choose a bivalent formulation if vaccinated, and for 82.2% it does not matter.

For 190 (33.7%) of the respondents, the availability of bivalent preparations will contribute to accelerating their decision to vaccinate against COVID-19. Among those with a completed basic regimen (without a booster dose), only 21.9% say they will be vaccinated quicker after the emergence of the new preparations. Among those who cannot decide on COVID-19 vaccination, 32.6% believe that bivalent preparations will speed up their decision.

In the subjective assessment, 117 (20.7%) of respondents believe that the implementation of bivalent preparations will increase interest in COVID-19 vaccination in Poland. A detailed summary is presented in Table 3.

## 4. Discussion

After more than a year of experience, we know that among other long-COVID consequences they contribute to a definite reduction in the severity of the disease, mortality and its sequelae [7,8,9]. However, due to dynamic changes within the virus, their effectiveness is diminishing [10,12,13,14]. In 2022, the scientists started the development of updated vaccine formula that will be able to target the Omicron variant In August 2022, the UK became the first country in the world to authorize the bivalent formulation for widespread use [18]. In Poland, despite many efforts, the vaccination rate is 59%, far below the European Union average of 66%. Likewise, with the booster dose, which was taken by 34% of those eligible [4]. Therefore, the purpose of the present study was to assess whether bivalent vaccination could increase the popularity of COVID-19 vaccination in Poland.

The results of the present study are not optimistic. Admittedly, 72.3% of respondents declared that given the opportunity to choose a formulation, they would opt for a bivalent formulation. However, this percentage is not high among the unvaccinated (8.9%) and those who have only completed the basic scheme (65.3%). Moreover, when asked about the impact of the availability of such formula on vaccination-related decisions, those without a booster dose, only 21.9% say that bivalent vaccination will accelerate their decision to take it. Among the unvaccinated, the percentage is only 5.1%. These people should now be the main target groups for vaccination because even though Omicron variant infections are characterized by milder courses and a lower risk of death, it still poses a health risk to the aforementioned individuals [21,22]. According to studies, unvaccinated people are at significantly higher risk of severe disease and death from COVID-19 [23]. Although primary vaccination induces immunity against the Omicron variant, this immunity declines with time. Those who have received a booster dose are less likely to develop a course of severe disease, but this level also declines over time [14,24,25,26].

Unfortunately, those who have not yet received a single dose of COVID-19 vaccination are unlikely to ever vaccinate, as confirmed in our study and previous reports [27]. Of those not yet vaccinated, 89% said they never planned to be vaccinated, and 11% would consider it in the future. It can be suspected that this group includes people who question the pandemic, the effectiveness of vaccination and downplay the severity of the COVID-19 disease [27,28]. Accordingly, the focus should be on the group of people who are still undecided. These people should become the target of education efforts, with prior identification of factors that discourage them from being vaccinated. While anti-vaccinationists already have an established opinion on vaccination and it is very difficult to convince them to change their attitude, in the case of doubters, a solid presentation of evidence can lead to a change in their attitude [29].

Another important aspect is that only 48.7% of respondents indicate that they are aware of bivalent vaccination, which may also affect the outcome. A study by Huoba Li et al. found that with increasing vaccination-related knowledge (education?), the willingness to receive it also increases [30]. In addition, the literature is still very poor in data on the efficacy and safety of these preparations, and as is known, the efficacy and safety of the used preparation are the most important predictors of willingness to vaccinate [5]. One study by Zhenhao Fang and Sidi Chen, which at the time of writing this manuscriptis in the preprint phase, showed that a booster dose of a bivalent vaccine induced an immune response to a greater extent [31]. In addition, a clinical trial conducted by Moderna on more than 400 volunteers also showed an eightfold increase in the level of neutralizing antibodies against the BA.1 variant compared to baseline. Equally important, the side effects that occurred were very similar to the original formulation. These were usually mild complaints that resolved spontaneously [32].

The author is aware of the limitations of the present study, which is undoubtedly the methodology of data collection using an online questionnaire. This method entails the impossibility of assessing the number of individuals that reached the survey but eventually did not complete it (i.e., the response rate). It is also important to note that online surveys are fraught with the risk of obtaining the results that depend on the self-selection of the survey group. To avoid this phenomenon, the questionnaire was distributed both within groups related to vaccination, COVID-19, and general forums, to diversify the group of respondents as much as possible. On the other hand, nowadays surveys using online questionnaires have become a common methodological tool for reaching a wide audience in a short time. Moreover, the analyzed group is not representative of Polish society. The overwhelming predominance of women, residents of large cities may affect the final results. In addition, among the analyzed respondents, the vast majority are already vaccinated, which is also significantly different from the average level of vaccination in Poland. However, to the best of the author’s knowledge, the present study is the first to assess Poles’ attitudes toward bivalent vaccination, which accounts for its strength and innovation.

In summary, the results of the present study do not inspire optimism that the implementation of bivalent vaccination will significantly elevate the interest rate regarding COVID-19 vaccination among previously unvaccinated individuals and those without a booster dose. Only 20 percent of those surveyed said that bivalent vaccines could increase the willingness to vaccinate. On the other hand, these vaccinations may be readily accepted by those who have already received 3 doses of vaccination, thereby strengthening their level of immunity against the currently dominant Omicron variant.

## 5. Conclusions

Bivalent vaccination is an important part of the counteraction against the ongoing COVID-19 pandemic; however, it is forecasted that its implementation will not significantly elevate the interest rate regarding the vaccination among those previously unvaccinated or only following the basic schedule. It is necessary to make further observations in this direction, to determine the public needs in terms of knowledge about the currently used preparations, and to implement appropriate campaigns that can elevate the vaccination-related interest and rate.

## Figures and Tables

**Table 1:** Characteristics of the study group.

Variable		N (%)
**Age M** **± SD**		36.6 ± 9.67
**Sex**	Male	117 (20.7)
	Female	447 (79.3)
**Place of residence**	Rural area	80 (14.2)
	City of up to 50,000 inhabitants	99 (17.5)
	City of 50,000–250,000 inhabitants	133 (23.6)
	City with more than 250,000 inhabitants	252 (44.7)
**Education**	Higher	487 (86.3)
	Secondary	77 (13.7)
	Other	0 (0.0)
**Relationship status**	Single	110 (19.5)
	Partnership	103 (18.3)
	Married	351 (62.2)
**Healthcare professional**	Yes	199 (35.3)
	No	365 (64.7)
**Chronic conditions**	Yes	254 (45.0)
	No	310 (55.0)

M—mean; SD—Standard deviation; N—number.

**Table 2:** Vaccination status and past vaccination history among respondents.

Variable		N (%)
**Vaccination against COVID-19**	Booster dose	390 (69.1)
	Basic vaccination scheme	95 (16.8)
	Incomplete scheme	0 (0.0)
	Unvaccinated	79 (14.1)
**COVID-19 vaccines taken (*n* = 485)**	Pfizer/BioNTech	311 (64.1)
	Moderna	39 (8.1)
	AstraZeneca	33 (6.8)
	Johnson&Johnson	18 (3.7)
	Mixed	84 (17.3)
**Adverse events following COVID-19 vaccination (*n* = 485)**	Severe	0 (0.0)
	Moderate	69 (14.2)
	Mild	276 (56.9)
	None	140 (28.9)
**Vaccination history**	Mandatory and recommended	295 (52.3)
	Only mandatory	231 (41.0)
	No	38 (6.7)
**Willingness to take the booster dose**	Yes, as soon as possible	299 (53.0)
	Yes, within a year	69 (12.2)
	Yes, but in a year or more	8 (1.4)
	I cannot decide	46 (8.2)
	No, but maybe in the future	43 (7.6)
	No, never	99 (17.6)

**Table 3:** Comparison of responses to questions assessing the impact of bivalent formulations on accelerating the decision to vaccinate, along with the preferred type of vaccine in relation to sociodemographic variables and previous vaccination status.

Variable		Influence on Vaccination Decision N (%)				Preferred Vaccination N (%)			
		Acceleration	Deferral	Does Not Matter	*p*	Bivalent	Monovalent	Does Not Matter	*p*
**Sex**	Male	33 (28.2)	12 (10.3)	72 (64.2)	<0.001	67 (57.3)	15 (12.8)	35 (29.9)	<0.001
	Female	157 (35.1)	3 (0.7)	287 (64.2)		341 (76.3)	7 (1.6)	99 (22.1)	
**Place of residence**	Rural area	29 (36.3)	4 (5.0)	47 (58.7)	0.691	54 (67.5)	4 (5.0)	22 (27.5)	<0.001
	City of up to 50,000 inhabitants	32 (32.3)	4 (4.0)	63 (63.7)		53 (53.5)	4 (4.0)	42 (42.5)	
	City of 50,000–250,000 inhabitants	45 (33.8)	3 (2.3)	85 (63.9)		103 (77.4)	7 (5.3)	23 (17.3)	
	City with more than 250,000 inhabitants	84 (33.3)	4 (1.6)	164 (65.1)		198 (78.6)	7 (2.8)	47 (18.6)	
**Education**	Higher	170 (34.9)	11 (2.3)	306 (62.8)	0.143	362 (74.3)	11 (2.3)	114 (23.4)	<0.001
	Secondary	20 (25.9)	4 (5.2)	53 (68.9)		46 (59.7)	11 (14.3)	20 (26.0)	
	Other	---	---	---		---	---	---	
**Relationship status**	Single	39 (35.5)	0 (0.0)	71 (64.5)	0.003	88 (80.0)	0 (0.0)	22 (20.0)	<0.001
	Partnership	22 (21.4)	8 (7.8)	73 (70.8)		58 (56.3)	11 (10.7)	34 (33.0)	
	Married	129 (36.8)	7 (2.0)	215 (61.2)		262 (74.6)	11 (3.1)	78 (22.3)	
**Healthcare professional**	Yes	75 (37.7)	3 (1.5)	121 (60.8)	0.178	159 (79.9)	4 (2.0)	36 (18.1)	0.007
	No	115 (31.5)	12 (3.3)	238 (65.2)		249 (68.2)	18 (4.9)	98 (26.9)	
**Chronic conditions**	Yes	88 (34.7)	0 (0.0)	166 (65.3)	0.001	204 (80.3)	7 (2.8)	43 (16.9)	<0.001
	No	102 (32.9)	15 (4.8)	193 (62.3)		204 (65.8)	15 (4.8)	91 (23.4)	
**Vaccination against COVID-19**	Booster dose	166 (42.6)	0 (0.0)	224 (57.4)	<0.001	339 (86.9)	4 (1.0)	47 (12.1)	<0.001
	Basic vaccination scheme	20 (21.9)	11 (11.6)	64 (67.4)		62 (65.3)	11 (11.6)	22 (23.1)	
	Unvaccinated	4 (5.1)	4 (5.1)	71 (89.8)		7 (8.9)	7 (8.9)	65 (82.2)	
**Vaccination history**	Mandatory and recommended	128 (43.4)	4 (1.4)	163 (55.2)	<0.001	261 (88.5)	4 (1.4)	30 (10.1)	<0.001
	Only mandatory	54 (23.4)	7 (3.0)	170 (73.6)		131 (56.7)	15 (6.5)	85 (36.8)	
	No	8 (4.1)	4 (10.5)	26 (68.4)		16 (42.1)	3 (7.9)	19 (50.0)	
**Willingness to take the booster dose**	Yes, as soon as possible	132 (44.2)	0 (0.0)	167 (55.8)	<0.001	289 (96.7)	0 (0.0)	10 (3.3)	<0.001
	Yes, within a year	36 (52.2)	0 (0.0)	33 (47.8)		63 (91.3)	0 (0.0)	6 (8.7)	
	Yes, but in a year or more	0 (0.0)	0 (0.0)	8 (100)		0 (0.0)	4 (50.0)	4 (50.0)	
	I cannot decide	15 (32.6)	0 (0.0)	31 (67.4)		35 (76.1)	3 (6.5)	8 (17.4)	
	No, but maybe in the future	7 (16.3)	7 (16.3)	29 (67.4)		21 (48.8)	4 (9.3)	18 (41.9)	
	No, never	0 (0.0)	8 (8.1)	91 (91.9)		0 (0.0)	11 (11.1)	88 (88.9)	

## Data Availability

The data presented in this study are available on request from the corresponding author.
